# *Streptococcus pneumoniae *stabilizes tumor necrosis factor α mRNA through a pathway dependent on p38 MAPK but independent of Toll-like receptors

**DOI:** 10.1186/1471-2172-9-52

**Published:** 2008-09-16

**Authors:** Trine H Mogensen, Randi S Berg, Lars Østergaard, Søren R Paludan

**Affiliations:** 1 Department of Infectious Diseases, Skejby Hospital – Aarhus University Hospital, DK-8200, Aarhus N, Denmark; 2 Institute of Medical Microbiology and Immunology, University of Aarhus, DK-8000, Aarhus C, Denmark

## Abstract

**Background:**

*Streptococcus pneumoniae *is a human pathogenic bacteria and a major cause of severe invasive diseases, including pneumonia, bacteremia, and meningitis. Infections with *S. pneumoniae *evoke a strong inflammatory response, which plays a major role in the pathogenesis of pneumococcal disease.

**Results:**

In this study, we have examined how *S. pneumoniae *affects expression of the inflammatory cytokine tumor necrosis factor (TNF) α, and the molecular mechanisms involved. Secretion of TNF-α was strongly induced by *S. pneumoniae*, which was able to stabilize TNF-α mRNA through a mechanism dependent on the viability of the bacteria as well as the adenylate uridylate-rich elements in the 3'untranslated region of TNF-α mRNA. The ability of *S. pneumoniae *to stabilize TNF-α mRNA was dependent on the mitogen-activated protein kinase (MAPK) p38 whereas inhibition of Toll-like receptor signaling via MyD88 did not affect *S. pneumoniae-*induced mRNA stabilization. P38 was activated through a pathway involving the upstream kinase transforming growth factor-activated kinase 1 and MAPK kinase 3.

**Conclusion:**

Thus, *S. pneumoniae *stabilizes TNF-α mRNA through a pathway dependent on p38 but independent of Toll-like receptors. Production of TNF-α may contribute significantly to the inflammatory response raised during pneumococcal infection.

## Background

*Streptococcus pneumoniae *is an important cause of severe invasive infections, including pneumonia, bacteremia, and meningitis [1,2]. Nasopharyngeal carriage of *S. pneumoniae *is common among children, but in most cases bacterial invasion into the lungs or bloodstream is prevented by local host defenses in the upper airways [3]. However, in a minority of individuals, pneumocooci gain access to deeper structures of the body and thereby cause invasive disease [3], which are almost uniformly fatal if not treated with antibiotics. Even in the presence of appropriate treatment, invasive pneumococcal disease bears a relatively poor prognosis [4]. Pneumococci are known to elicit a very potent inflammatory response [1,5], a key component of which is the production of cytokines and chemokines, which participate in the elimination of bacteria but may also result in undesirable excessive immunological responses, which may be harmful to the host [1].

The cytokine tumor necrosis factor (TNF)-α is produced by macrophages and dendritic cells as a primary response to infections and tissue damage, and is constitutively expressed in several autoimmune diseases [6]. TNF-α plays an important role in activation and recruitment of leukocytes to inflamed tissue [6], and has been demonstrated to be involved in the host-defense against a number of important human pathogens, including *S. pneumoniae *[7-9]. However, TNF-α is also associated with excessive inflammation and immunopathology in infections and autoimmune diseases, and specifically, TNF-a has been suggested to be involved in breakage of the blood-brain barrier during development of hematogenous pneumococcal meningitis [10].

Production of TNF-α is regulated both at the level of transcription, mRNA stability, and translation [11-13]. The regulation of mRNA stability and translation is mediated largely through adenylate uridylate (AU)-rich elements (ARE)s present in the 3'-untranslated region (UTR) of mRNA, which are targeted by ARE-binding proteins able to affect mRNA stability and translation [13,14]. The importance of AREs in regulation of TNF-α production is evidenced by spontaneous development of chronic inflammatory arthritis and Crohn's-like inflammatory bowel disease in transgenic mice devoid of AREs in mRNA encoding TNF-α [12].

The inflammatory response to infection is triggered by pattern recognition receptors (PRR)s, which recognize evolutionarily conserved pathogen-associated molecular patterns (PAMP)s and activate intracellular signaling, thereby up-regulating expression of genes with inflammatory activities, including TNF-α [15]. The Toll-like receptors (TLR)s constitute an important class of PRRs, and TLR2, 4, and 9 have been shown to recognize pneumococcal PAMPs [3,16-20]. The vast majority of TLR downstream signaling proceeds through the cytoplasmic adaptor protein MyD88, which is central for activation of the transcription factor nuclear factor (NF) κB, and the mitogen-activated protein kinase (MAPK) pathway, being involved in both transcriptional and post-transcriptional responses [21]. However, several studies suggest that pneumococcal recognition and pathogenesis may be at least partly independent of TLR signaling [22,23]. Another class of PRRs involved in recognition of *S. pneumoniae *is the nucleotide-binding oligomerization domain (NOD) proteins, which are intracellular PRRs [24]. The NOD2 protein recognizing muramyl dipeptide has been reported to be important for activation of NF-κB during infection with *S. pneumoniae *[25].

In this study, we have investigated the role of mRNA stabilization in TNF-α production induced by *S. pneunomiae*, including the signaling mechanisms involved. Here we report that live *S. pneunomiae *strongly stabilizes TNF-α mRNA by a mechanism dependent on the AREs in the 3'-UTR of the mRNA. Importantly, increased TNF-α mRNA stability induced by *S. pneunomiae *was not mediated by TLRs but was dependent on the p38 MAPK, which was activated through a pathway involving transforming growth factor-activated kinase (TAK) 1 and MAPK kinase (MKK) 3.

## Results

### *S. pneumoniae *induces TNF-α production and stabilizes TNF-α mRNA through AREs in the 3'-UTR

**Figure 1:**
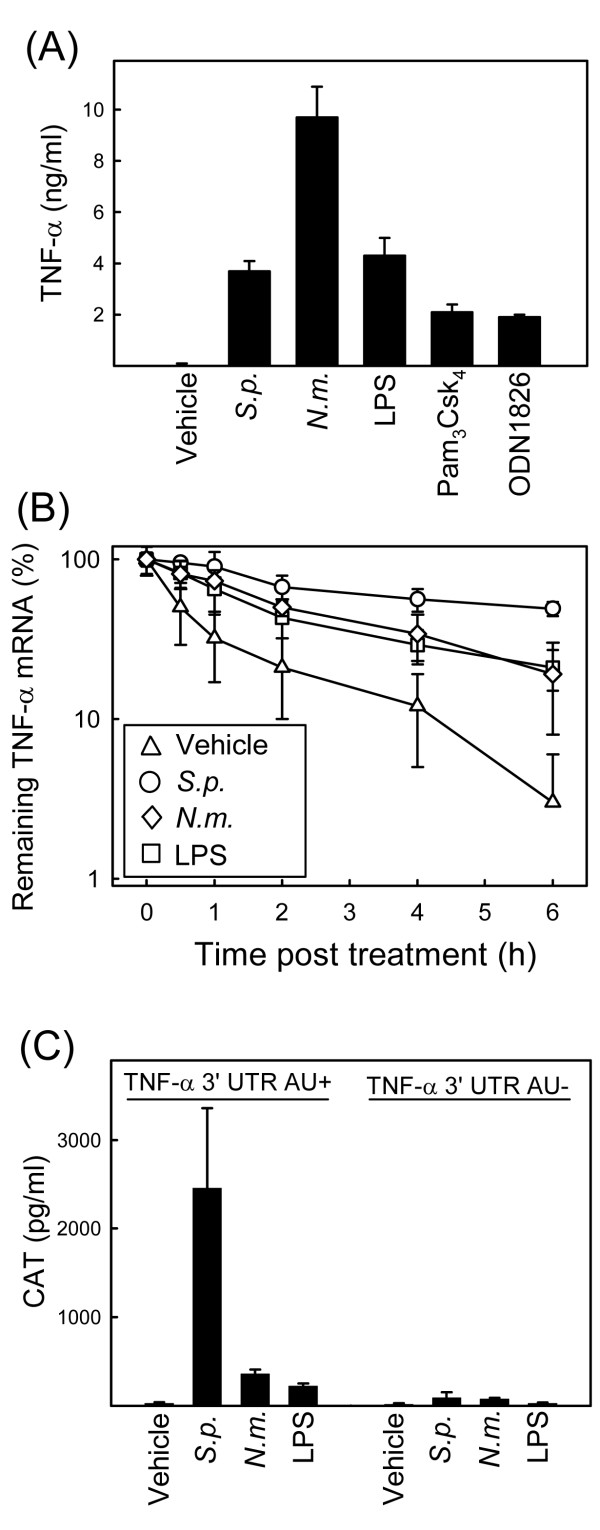
***S. pneumoniae *induces TNF-α production and stabilizes TNF-α mRNA through AREs in the 3'-UTR**. (A) C57BL/6 macrophages were stimulated with vehicle, *S. pneumoniae*, *N. meningitidis *(5 × 10^7 ^bacteria/ml), LPS (100 ng/ml), Pam3Csk4 (200 ng/ml), or ODN1826 (1 μM). Supernatants were harvested 18 h later, and TNF-α was measured by ELISA. (B) C57BL/6 macrophages were treated with 1 μM actinomycin D 15 min prior to treatment with vehicle, *S. pneumoniae*, *N. meningitidis *(both 5 × 10^7 ^bacteria/ml), or LPS (100 ng/ml). The cells were lysed at the indicated time points and Total RNA was harvested. TNF-α and β-actin mRNAs were detected by qPCR, and normalized ratios were calculated. The data is shown as percent remaining TNF-α mRNA compared to untreated control. (C) RAW-TNF-AU+ and RAW-TNF-AU÷ cells were seeded and treated with vehicle, *S. pneumoniae*, *N. meningitidis *(both 5 × 10^7 ^bacteria/ml), or LPS (100 ng/ml). Total cell lysates were harvested 20 h later and CAT was measured by ELISA. Similar results were obtained in 2–4 independent experiments. The data are shown as means +/- SEM.

Given the important role of the inflammatory response in the pathogenesis of streptococcal diseases [1], we were interested in studying mechanisms involved in expression of inflammatory cytokines during infection with *S. pneumoniae*. In a first set of experiments, we measured expression of TNF-α in murine macrophages after treatment with *S. pneumoniae*, *N. meningitidis *or pure ligands for TLR2 (Pam3Csk4), TLR4 (LPS), and TLR9 (ODN1826). As expected, cells responded to addition of bacteria or TLR ligands by secreting high amounts of TNF-α (Fig. 1A). LPS has been reported to stimulate TNF-α expression by both activating gene transcription, stabilizing the TNF-α mRNA, and supporting translation [11-13]. To examine whether *S. pneumoniae *stabilized TNF-α mRNA, we treated cells with actinomycin D, which prevents *de novo *transcription of mRNA, and stimulated the macrophages with *S. pneumoniae, N. meningitidis *or LPS. At the indicated time-points post-stimulation, RNA was harvested and TNF-α mRNA levels were determined by real-time PCR. As shown in Fig. 1B, addition of actinomycin D led to a rapid decline in the levels of remaining TNF-α mRNA in cells receiving mock treatment. However, treatment with *S. pneumoniae *profoundly augmented the half-life of TNF-α mRNA (from about 30 min to about 6 hours). LPS and *N. meningitidis *also stabilized the TNF-α messenger but not to the same extent as *S. pneumoniae*. To examine whether the AREs of the TNF-α mRNA were required for the observed effect, we turned to a cell system derived from RAW264.7 cells, consisting of two cell lines stably expressing a reporter gene (chloramphenicol acetyl transferase, CAT), transcribed from a constitutive promoter. In one of the cell lines (RAW TNF-α 3'-UTR AU+) the 3'-UTR of the mRNA encoding the reporter gene is derived from the wild-type TNF-α mRNA, and in the other cell line (RAW TNF-α 3'-UTR AU÷) the AREs of the TNF-α 3'-UTR are mutated. When the RAW TNF-α 3'-UTR AU+ cell line was incubated in the presence of *S. pneumoniae*, a very strong increase in CAT levels was observed (Fig. 1C), whereas LPS and also *N. meningitidis *affected accumulation of CAT protein only to a more modest extent. Interestingly, the effect of *S. pneumoniae *on accumulation of CAT was largely abrogated in the RAW TNF-α 3'-UTR AU÷ cell line. Thus, *S. pneumoniae *stimulates production of TNF-α and stabilizes TNF-α mRNA through a mechanism involving the AREs in the 3'-UTR of the mRNA.

### Stabilization of TNF-α mRNA by *S. pneumoniae *is dependent on the viability of the bacteria

**Figure 2:**
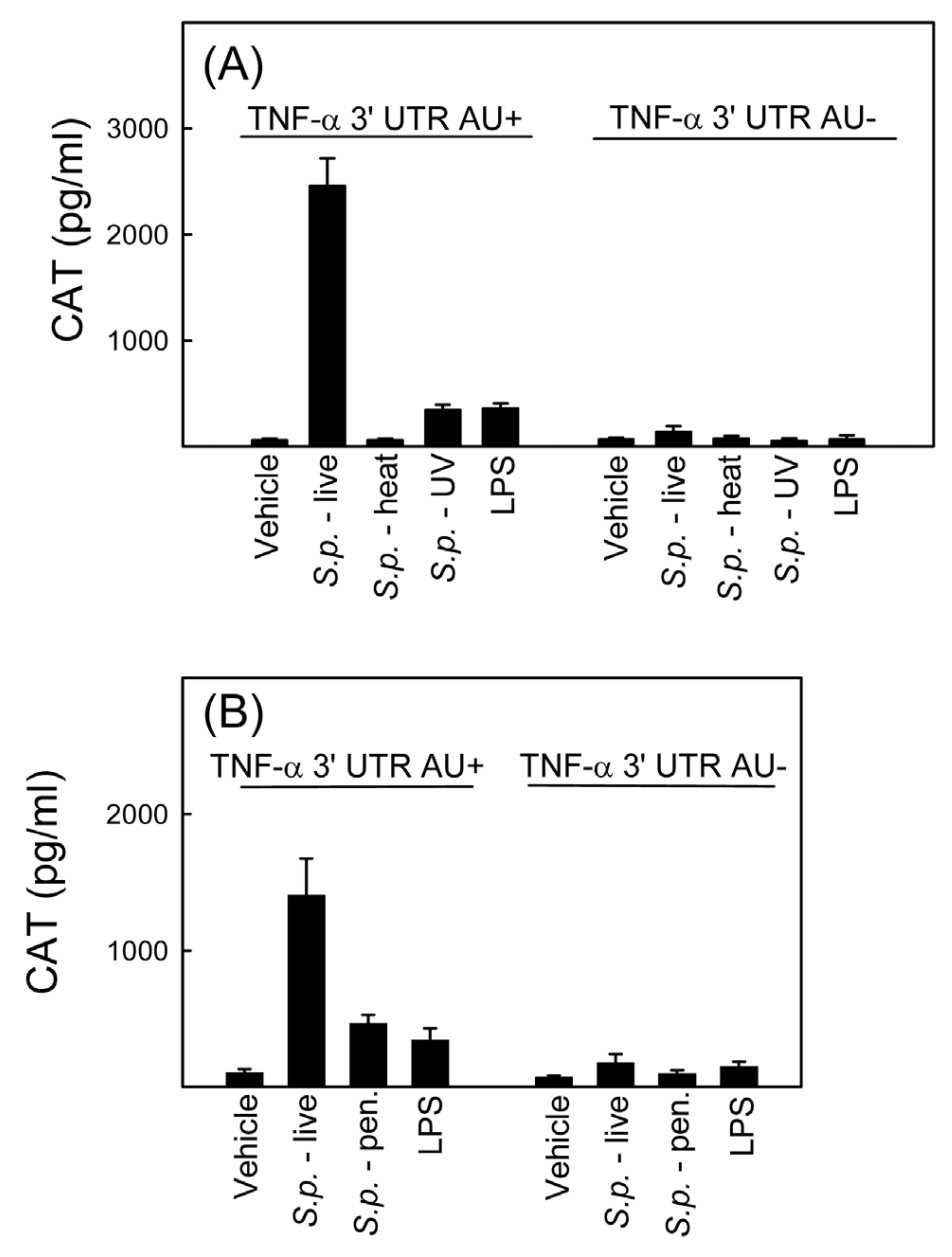
**Stabilization of TNF-α mRNA by *S. pneumoniae *is dependent on the viability of the bacteria**. RAW-TNF-AU+ and RAW-TNF-AU÷ cells were seeded and treated with vehicle, live *pneumoniae *(5 × 10^7 ^bacteria/ml), or LPS (100 ng/ml) as well as bacteria (5 × 10^7 ^bacteria/ml) killed by either (A), heat, UV, or (B) penicillin treatment. Total cell lysates were harvested 20 h later and CAT was measured by ELISA. The data is shown as means +/- SEM. Similar results were obtained in 2–3 independent experiments.

We next examined the pneumococcal requirement for mediating TNF-α mRNA stability. To this end, we compared the ability of live *versus *killed bacteria to induce CAT expression in the RAW TNF-α 3'-UTR AU+/AU- cell lines (Fig. 2A and 2B). As also shown in Fig. 1C, *S. pneumoniae *induced accumulation of CAT, and this was dependent on the 3'-UTR AREs (Fig. 2). Interestingly, *S. pneumoniae *killed either by heat-inactivation, UV light or penicillin displayed reduced ability to induce CAT expression, with the reduction ranging from almost 100% (heat-inactivated bacteria) to about 65% (penicillin-killed bacterial). Thus, stabilization of TNF-α mRNA by *S. pneumoniae *is dependent on the viability of the bacteria.

### *S. pneumoniae *mediates TNF-α mRNA stability independent of TLRs

**Figure 3:**
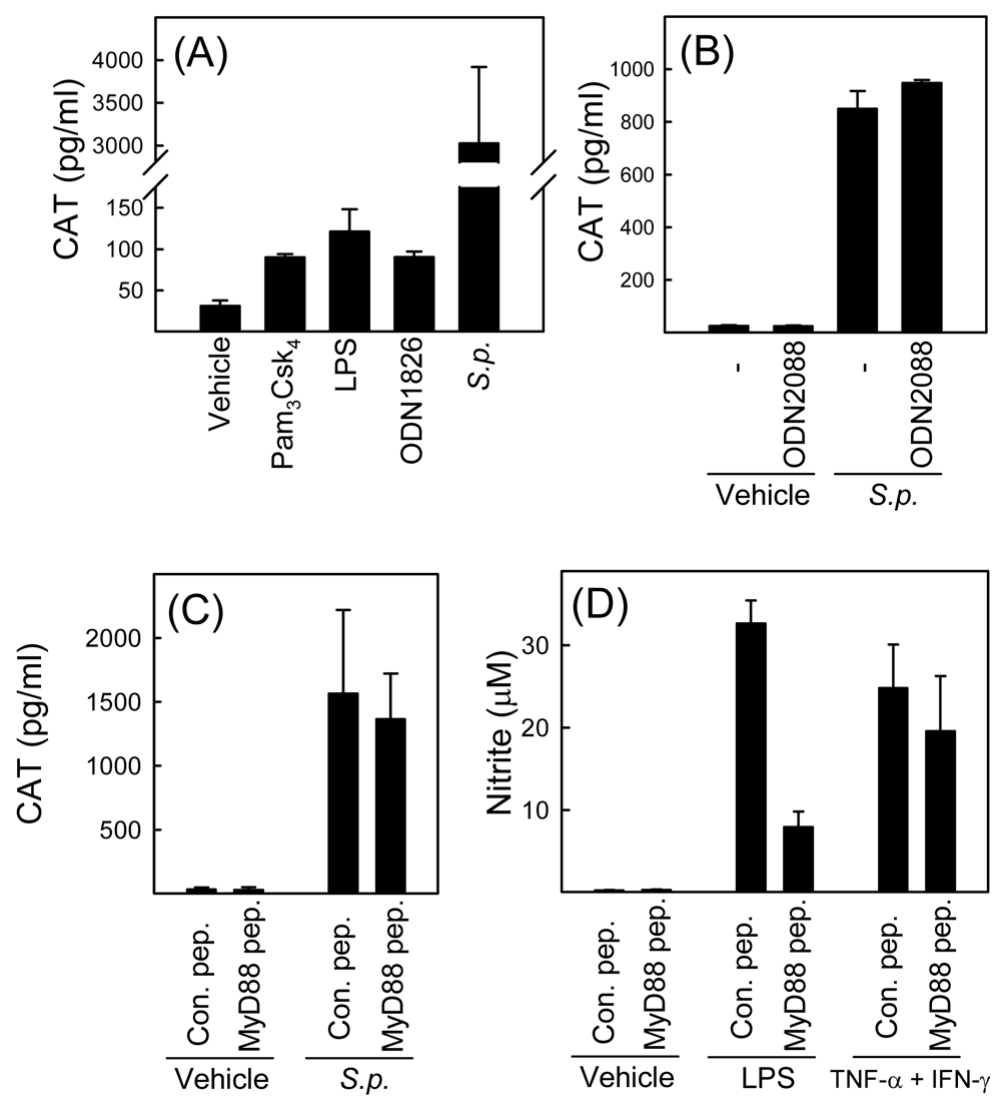
***S. pneumoniae *mediates TNF-α mRNA stability independent of TLRs**. (A) RAW-TNF-AU+ cells were treated with vehicle, *S. pneumoniae *(5 × 10^7 ^bacteria/ml), LPS (100 ng/ml), Pam3Csk4 (200 ng/ml), or ODN1826 (1 μM). Total cell lysates were harvested 20 h later and CAT was measured by ELISA. (B) RAW-TNF-AU+ cells were treated with the TLR9 antagonist ODN2088 (3 μM) 15 min prior to addition of vehicle or *S. pneumoniae *(5 × 10^7 ^bacteria/ml) to the cell cultures. Total cell lysates were harvested 20 h later and CAT was measured by ELISA. (C and D) RAW-TNF-AU+ cells were treated with a MyD88 inhibitor peptide or a control peptide for 24 h before addition of (C) vehicle or *S. pneumoniae *(5 × 10^7 ^bacteria/ml) or (D) LPS (100 ng/ml) or TNF-α (25 ng/ml) plus IFN-γ (10 ng/ml). (C) Total cell lysates were harvested 20 h later and CAT was measured by ELISA. (D) Supernatants were harvested 36 h post stimulation, and nitrite was measured by Griess assay. Similar results were obtained in 2–3 independent experiments. The data are shown as means +/- SEM.

The *S. pneumoniae *SK1013 strain used in this study has previously been demonstrated to be recognized by TLR2 and 9 [16], with the former recognizing bacterial peptidoglycan and lipoteichoic acid and the latter recognizing bacterial DNA [16-18,26]. We have previously demonstrated that TLR9 recognizes live but not heat-killed *S. pneumoniae *[16] wherefore the data shown in Fig. 2 prompted us to examine the role of TLR9 in stabilization of TNF-α mRNA. We first examined how pure TLR agonists affected CAT expression in the RAW-TNF-α 3'-UTR AU+ cell line and compared this with the response evoked by live *S. pneumoniae*. The agonists for TLR2, 4, and 9 all enhanced CAT expression but to a rather moderate extent compared to what was observed in cells receiving live *S. pneumoniae *(Fig. 3A). To directly assess the role of TLR9, we treated cells with the TLR9 antagonist ODN2088 prior to addition of *S. pneumoniae *and measured CAT expression in lysates at later time points. Although ODN2088 inhibited induction of TNF-α by the TLR9 agonist ODN1826 by more than 95% (data not shown), the presence of the TLR9 antagonist had no effect on CAT expression induced by *S. pneumoniae *(Fig. 3B), thus suggesting that mRNA stabilization by *S. pneumoniae *is independent of TLR9.

To more broadly examine the role of TLRs in stabilization of TNF-α mRNA during *S. pneumoniae *infection, we pretreated cells with a cell-permeable MyD88 inhibitory peptide or an inactive control peptide prior to addition of bacteria. However, this treatment did not affect *S. pneumoniae*-induced CAT expression (Fig. 3C), although the inhibitory peptide did indeed inhibit TLR signaling both strongly and specifically (Fig. 3D). Thus, the ability of *S. pneumoniae *to stabilize TNF-α mRNA seems to be independent of bacterial signaling through TLRs.

### P38 MAPK is important for stabilization of TNF-α mRNA by *S. pneumoniae*

**Figure 4:**
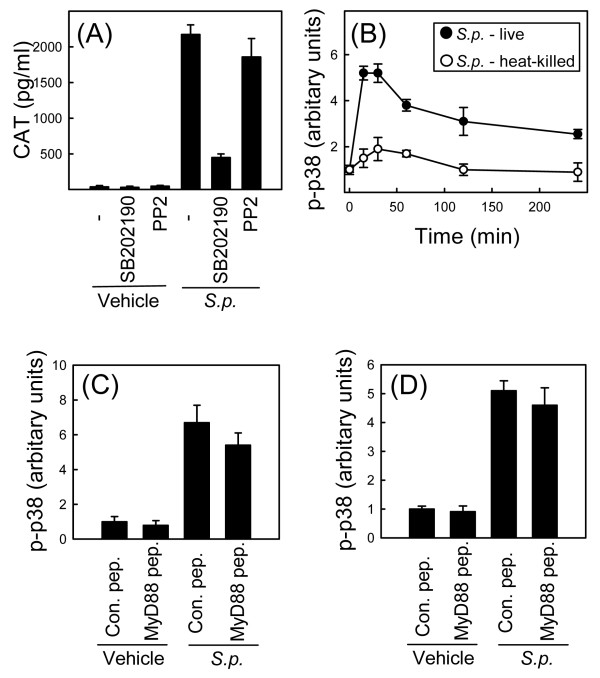
**P38 MAPK is important for stabilization of TNF-α mRNA by *S. pneumoniae***. (A) RAW-TNF-AU+ cells were treated with the inhibitors SB202190 (p38 and RIP2) (5 μM) and PP2 (Src and RIP2) (500 nM) 15 min prior to addition of vehicle or *S. pneumoniae *(5 × 10^7 ^bacteria/ml) to the cell cultures. Total cell lysates were harvested 20 h later and CAT was measured by ELISA. The data is shown as means +/- SEM. (B) RAW264.7 cells were treated with live or heat-killed *S. pneumoniae *(5 × 10^7 ^bacteria/ml) for the indicated amount of time, and whole cell lysates were isolated. Phosphorylation of p38 was measured by Luminex. The data is shown as means +/- SEM. (C and D) RAW264.7 (C) and C57BL/6 macrophages (D) were incubated with a MyD88 inhibitor peptide or a control peptide for 24 h before addition of vehicle or *S. pneumoniae *(5 × 10^7 ^bacteria/ml). Whole cell lysates were harvested 30 min later and phosphorylation of p38 was measured by Luminex. The data is shown as means +/- SEM. Similar results were obtained in 2–3 independent experiments.

The signaling pathways responsible for mediating mRNA stability have recently been studied extensively, and it appears that P38 MAPK plays a central role in this process [13]. To examine the role of p38 in stabilization of TNF-α mRNA in response to *S. pneumoniae *infection, we treated the RAW TNF-α 3'-UTR AU+ cell line with the p38 inhibitor SB202190 15 min prior to addition of *S. pneumoniae*. CAT was subsequently measured in cell lysates. As shown in Fig. 4A, the strong elevation of CAT protein levels observed after infection with live *S. pneumoniae *was significantly inhibited by the presence of SB202190. It has been reported that the p38 MAPK inhibitor SB203580, which is structurally very similar to SB202190, not only inhibits p38 but also the kinase receptor-interacting protein (RIP) 2 [27], which plays an important role in signaling downstream of the *S. pneumoniae*-activated PRR NOD2 [27]. Therefore, we also examined the effect of another RIP2 inhibitor, PP2 [27], on induction of CAT protein in our reporter system However, as illustrated in Fig. 4A, no effect of PP2 treatment towards bacteria-induced CAT production was observed.

In Fig. 2 we demonstrated that live but not heat-killed *S. pneumoniae *mediated stabilization of TNF-α mRNA. Therefore, we hypothesized that live versus heat-killed *S. pneumoniae *may differentially activate p38. RAW264.7 cells were stimulated with live or heat-killed *S. pneumoniae *for the indicated time intervals, whole-cell lysates were harvested and the levels of phosphorylated p38 were measured. Indeed, the results showed that live bacteria potently stimulated phosphorylation of p38, heat-killed bacteria were not able to elicit such a response (Fig. 4B).

To more thoroughly study the role of p38 in *S. pneumoniae*-mediated mRNA stabilization, we wanted to examine how inactivation of TLR signaling, which did not affect TNF-α mRNA stabilization (Fig. 3), might influence p38 activation. Therefore, we pretreated RAW264.7 cells and primary macrophages with the MyD88 inhibitory peptide or an inactive control peptide prior to addition of *S. pneumoniae*. The cells were lysed 30 min post-infection and levels of phospho-p38 were measured. In both cell types we observed strong activation of p38 in response to *S. pneumoniae *treatment and this was independent of the presence of the MyD88 inhibitory peptide (Fig. 4C and 4D). Thus, stabilization of TNF-α mRNA by *S. pneumoniae *is dependent on p38, and this kinase is activated by live bacteria through a mechanism independent of TLRs.

### P38 mediates mRNA stabilization in response to *S. pneumoniae *independently of the MAPK-activated protein kinases MK2, MSK1/2, and MNK

P38-mediated mRNA stabilization can occur through several mechanisms, including involvement of members of the MAPK-activated protein kinase family, in particular MK2 [28-30]. To examine the role of this family of kinases in stabilization of TNF-α mRNA, we compared macrophages from C57BL/6 *versus *MK2-/- or MSK1/2-/- mice with respect to stabilization of endogenous TNF-α mRNA after challenge with *S. pneumoniae*. Furthermore, we also examined the effect of the Mnk inhibitor CGP57380 on production of CAT protein in the reporter cell line system. In all cases, no role for the MAPK-activated protein kinases was found (data not shown).

### Activation of p38 MAPK in response to *S. pneumoniae *is dependent on TAK1 and involves MKK3

**Figure 5:**
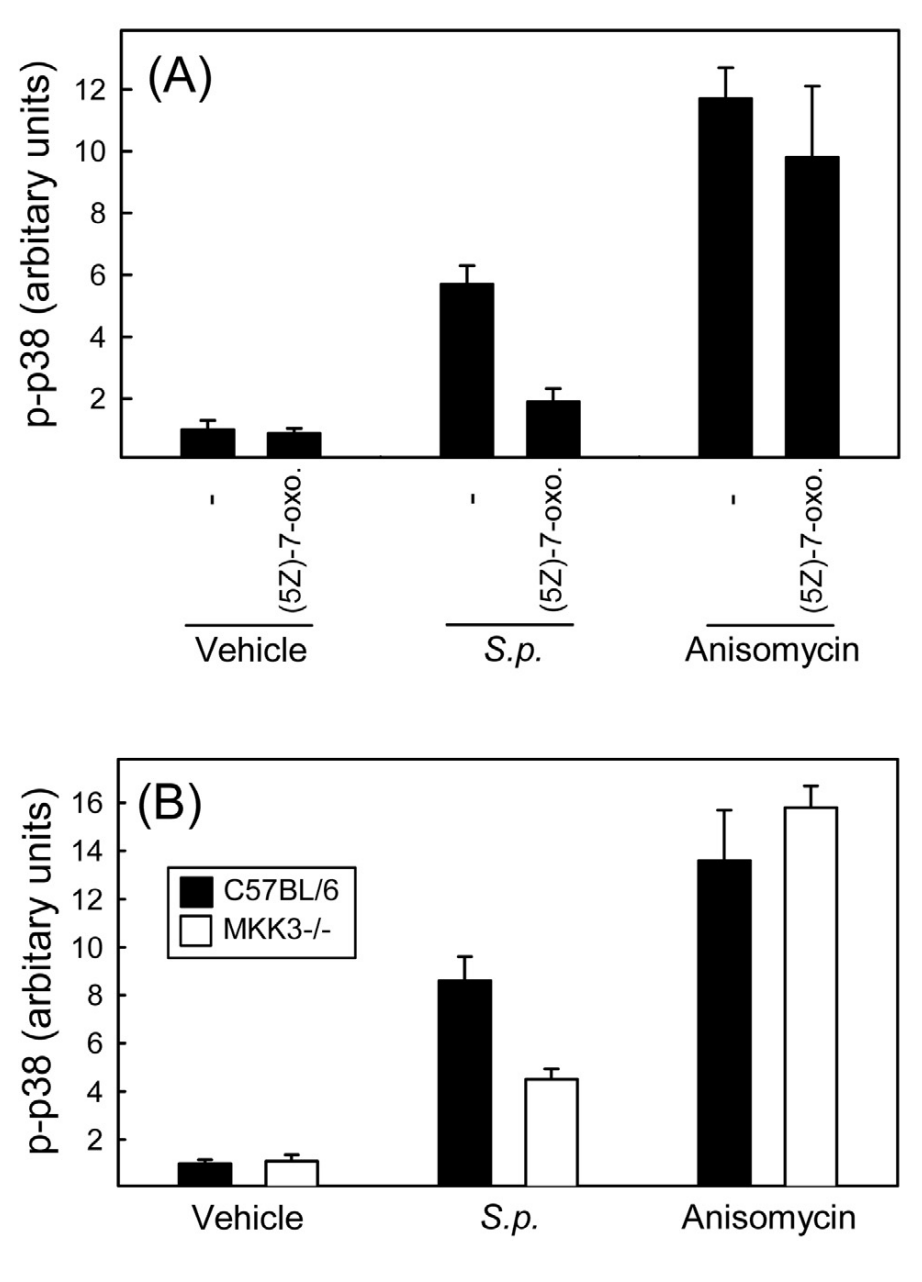
**Activation of p38 MAPK in response to *S. pneumoniae *is dependent on TAK1 and involves MKK3**. (A) RAW264.7 cells were seeded in triplicate cultures and left overnight to settle. Fifteen min prior to stimulation, some wells received 1 μM of (5*Z*)-7-oxozeaenol. The cells were stimulated with *S. pneumoniae *(5 × 10^7 ^bacteria/ml) or anisomycin (100 ng/ml) for 30 min, and whole-cell lysates were prepared. (B) Spleen cells from C57BL/6 and MKK3-/- mice were seeded in triplicates and left overnight to settle. The cells were stimulated with *S. pneumoniae *(5 × 10^7 ^bacteria/ml) or anisomycin (100 ng/ml) for 30 min, and whole-cell lysates were prepared. Phosphorylation of p38 MAPK was measured by Luminex, and data is presented as means +/- SEM. Similar results were obtained in 2 independent experiments.

In order to gain more insight into the mechanism of p38 activation during infection with *S. pneumoniae*, we first examined the role of TAK1, which has been ascribed an important role in this process by activating a number of different pathways [31-33]. RAW264.7 cells were seeded and treated with the TAK1 inhibitor (5*Z*)-7-oxozeaenol 15 min prior to stimulation with *S. pneumoniae *or anisomycin. Lysates were prepared 30 min post-stimulation, and levels of phospho-p38 were measured. As shown in Fig. 5A, both *S. pneumoniae *and anisomycin potently induced phosphorylation of p38. Whereas the TAK1 inhibitor did not affect activation of p38 in response to anisomycin, the ability of *S. pneumoniae *to induce p38 phosphorylation was abolished in the presence of the TAK1 inhibitor.

MKK3, as well as MKK4, and 6 are p38 upstream kinases and can be activated through TAK1 [34]. To evaluate the role of MKK3 in the *S. pneumoniae*-induced p38 activation pathway, we compared the ability of spleen cells from C57BL/6 and MKK3-/- mice to activate p38 in response to *S. pneumoniae *treatment. Anisomycin was included as a control, and was found to trigger p38 activation independently of MKK3 (Fig. 5B). By contrast, MKK3-/- cells responded to *S. pneumoniae *with significantly reduced, although not abolished, activation of p38. Taken together, these data demonstrate that *S. pneumoniae *activates p38 through a TLR-independent pathway involving the upstream kinases TAK1 and MKK3.

## Discussion

In this work we demonstrate that *S. pneumoniae *mediates stabilization of TNF-α mRNA in macrophages by a mechanism dependent on the AREs in the 3'-UTR of TNF-α mRNA. The pathway stimulating this mechanism is independent of TLR signaling and involves TAK1, MKK3, and p38 MAPK.

An important finding of this work is that recognition systems independent of TLRs are responsible for triggering *S. pneumoniae*-induced signal transduction leading to stabilization of TNF-α mRNA. Here we did not identify an alternative PRR responsible for activating the p38-dependent signal. It has been reported that *S. pneumoniae *is recognized by NOD2, which does indeed signal to p38 [24]. However, since inactivated *S. pneumoniae *should retain the capacity to activate NOD2, and we found that the RIP2 inhibitor PP2 did not affect expression of the CAT reporter, our data suggest that the observed stabilization of TNF-α mRNA by live *S. pneumoniae *does not depend on NOD2. In addition, there is evidence that other receptor systems may be involved in shaping the inflammatory cytokine response during bacterial infections. For instance, it has been reported that *Borrelia burgdorferi *can induce intracellular signaling and inflammatory gene expression independent of TLRs through direct binding to integrin α3 and β1 [35].

The present finding of only live *S. pneumoniae *being able to induce TNF-α mRNA stability suggests that a product or activity of the bacterial life cycle is required to trigger the p38-dependent signal mediating mRNA stability. Similar findings have been previously reported for other bacteria, since Kumar *et al*. demonstrated that live as opposed to heat-killed *Staphylococcus aureus *were able to stimulate phosphorylation of the MAPKs p38 and JNK, as well as the NF-κB inhibitory protein IκB [36]. In that study, however, the effects of live bacteria may have been attributable to bacterial exoproducts, since the conditioned medium of *S. aureus *also stimulated the signalling pathways [36].

The ability of *S. pneumoniae *to induce mRNA stabilization was abrogated in the presence of the p38 MAPK inhibitor SB202190, suggesting a role for this kinase. In addition, the signaling pathway to p38 was entirely dependent on TAK1 and partially dependent on MKK3. P38 MAPK is a well-described mediator of mRNA stabilization affecting the activity of a number of ARE-binding proteins [13,37]. For instance, p38 activates the kinase MK2, which phosphorylates tristetraprolin (TTP), an ARE-binding protein with mRNA-destabilizing activity [28]. This phosphorylation event decreases the ARE affinity of TTP, hence leading to increased mRNA stability. Another mechanism involves HuR, which is induced by P38 MAPK and binds to the AREs, thus stabilizing TNF-α mRNA [29,30]. In this work we did not systematically investigate the molecular mechanism of mRNA stabilization in response to *S. pneumoniae *infection, although we did find that it was independent of the MAPK-activated protein kinases MK2, MSK1/2, and MNK.

## Conclusion

Here we report that live *S. pneumoniae *stabilizes TNF-α mRNA and that this is dependent on the AREs in the 3'-UTR of the mRNA. The bacteria-induced signals mediating this function were not transduced from TLRs but were dependent on the p38 MAPK, the upstream activation of which was dependent on TAK1 and MKK3. Given the important role of TNF-α in the inflammatory response, the present study provides new insight into the mechanisms that govern production of inflammatory mediators in macrophages during pneumococcal infection.

## Methods

### Mice and cells

C57BL/6, MK2-/-, MSK1/2-/-, MKK3-/- mice were obtained from Taconic (Laven, Denmark), professor Mathias Gaestel (Hannover, Germany), professor Simon Arthur (Dundee, UK), and professor Richard A Flavell (Yale, USA), respectively, and bred in the animal facility of The Faculty of Health Sciences, University of Aarhus, under pathogen-free conditions. Peritoneal macrophages were harvested by lavage of the peritoneal cavity with PBS supplemented with 5% LPS-free heat-inactivated fetal calf serum (FCS) (Cambrex, Baltimore, MD, USA) and 400 μl heparin (Leo Pharma, Copenhagen, Denmark) per 100 ml. RAW264.7 macrophage-like cells and the derived cell lines RAW TNF-α 3' untranslated region (UTR) AU+ and RAW TNF-α 3'UTR AU÷ [38-40] were maintained in DMEM supplemented with 5% FCS, antibiotics and for the latter 2 cell lines also with 500 μg/ml G418 (Roche, Basel, Switzerland). For experiments, primary macrophages were seeded in 96-well or 6-well tissue plates in RPMI 1640 medium containing 5% FCS at a density of 3.0 × 10^5^and 6.0 × 10^6 ^cells per well, respectively. RAW264.7 and derived cell lines were seeded in 96-well or 6-well tissue plates at a density of 1.0 × 10^5 ^and 6.0 × 10^6 ^cells per well, respectively. After seeding, cells were left overnight to settle before further treatment.

### Bacteria

The bacteria used were the *S. pneumoniae *strain SK1013 serotype 4 (TIGR4) and the *N. meningitidis *strain NGO93 serogroup B, serotype 15:P1.2, MLEE cluster I1, ET76. The bacteria were grown overnight in Brain Heart Infusion broth with 10% Levinthal broth (Statens Serum Institute, Copenhagen) reaching a concentration of 18.0 ± 2.2 × 10^8 ^bacteria per ml as determined in a Thoma counting chamber. For stimulation, 300 μl and 10 μl from this stock was added to the cell cultures in 6- and 96-well plates, respectively, reaching final volumes of 3 ml and 100 μl, respectively. The bacteria were heat-inactivated by incubation of the cultures for 30 min at 65°C. To UV-inactivate the bacteria, the cultures were exposed to UV light for 10 min, and for killing of bacteria with penicillin, the cultures were incubated for 2 h with 1.0 μg/ml of the antibiotics.

### Reagents

The TLR agonists Pam3Csk4, LPS, and ODN1826 as well as the TLR9 antagonist ODN2088 were obtained from InvivoGen (San Diego, CA, USA). The MyD88 Homodimerization Inhibitory Peptide was purchased from IMGENEX (San Diego, CA, USA). Actinomycin D, anisomycin, SB202190, and PP2 were from Calbiochem (San Diego, CA, USA). The TAK1 inhibitor (5*Z*)-7-oxozeaenol was purchased from AnalytiCon Discovery GmbH. The cytokines TNF-α, and interferon (IFN)-γ were from R&D Systems (Minneapolis, MN, USA). The Mnk inhibitor CGP57380 was kindly provided by Hermann Gram (Novatis).

### TNF-α ELISA

For measurement of TNF-α. an ELISA duoset from R&D Systems was used, following recommendations of the manufacturer. The concentrations of coating Ab and detection Ab were 0.8 and 0.15 mg/ml, respectively. The ELISA was able to detect TNF-α from 15 to 2000 pg/ml.

### Purification of RNA and Real-time PCR

Total RNA was extracted with TRIzol (Invitrogen, Carlsbad, CA, USA) according to the recommendations of the manufacturer. Briefly, cells were lysed in TRIzol, and chloroform was added, followed by phase separation by centrifugation. RNA was precipitated with isopropanol and pelleted by centrifugation. Pellets were washed with 80% ethanol and re-dissolved in RNase-free water. For cDNA generation, 1 μg of RNA was subjected to reverse transcription (RT) with oligo(dT) as primer and Expand Reverse Transcriptase (both from Roche). Prior to RT-PCR, RNA was treated with DNase I (Ambion, Austin, TX, USA) to remove any contaminating DNA, the absence of which was confirmed in control experiments where the reverse transcriptase enzyme was omitted (data not shown). To measure the relative amount of TNF-α mRNAs, amplification of sample cDNA was monitored with the fluorescent DNA binding dye SYBR (QIAGEN). The PCR primers used in this study were: TNF-forward, TGGGAGTAGACAAGGTACAACCC; TNF-reverse, AGAGGGAAATCGTGCGTGAC; β-actin-forward, GCTCCCCGGGCTGTATTCC; β-actin-reverse, CTCTCTTGCTCTGGGCCTCGT). The data on TNF-α were normalized to β-actin, and presented as normalized ratio.

### Measurement of CAT levels

The cells were seeded and treated as described for the appropriate amount of time. For cell lysis and measurement of CAT levels the CAT ELISA kit from Roche (Basel, Switzerland) was used.

### Preparation of whole cell extracts

To assay for levels of total and phosphorylated p38, cells were seeded in 6-well plates as described above and stimulated with bacteria as specified in the text. At different time points post-stimulation, cells were lysed using the Bio-Plex Cell Lysis Kit (Bio-Rad, Hercules, CA, USA) according to the recommendations of the manufacturer. Briefly, the cells were washed with 3 ml Cell Wash Buffer per well and treated with 1 ml Lysing Solution supplemented with PMSF followed by incubation for 20 min at 4°C. The suspension was centrifuged at 4500 × g for 20 min at 4°C and supernatants were harvested as whole-cell extracts.

### Luminex

The levels of total and phosphorylated p38 were measured using the Luminex Technology™ and kits purchased from Bio-Rad. Briefly, the filter plate was washed with assay buffer and freshly vortexed antibody-conjugated beads were added to each well. The plate was washed with assay buffer and samples (45 μg of protein in 50 μl lysis buffer) were added. After a brief shake (30 sec at 1.100 rpm), the plate was incubated with shaking (300 rpm) overnight at 4°C. After a wash-step, detection antibody was added to each well, and the plate was shaken and incubated shaking (300 rpm) at room-temperature in the dark for 45 min to 2 h. Subsequently, the plate was washed and incubated for 10 min with 50 μl of a streptavidine-PE solution with shaking (30 sec at 1.100 rpm, 10 min 300 rpm). Finally the plate was washed and 125 μl of assay buffer was added to each well and the plate was shaken for 10 sec at 1100 rpm and read immediately on the Bio-Plex reader.

### Nitrite determination

Nitrite is generated by the rapid oxidation of NO. It is stable and its accumulation in the culture medium reflects the amount of NO produced. To assay nitrite we used the Griess reaction. Aliquots of 100 μl culture supernatants were mixed with equal volumes of Griess reagent (equal volumes of 0.1% *N*-(1-naphthyl)-ethylenediamine dihydrochloride and 1% *p*-aminobenzene sulphanilamide diluted in 2.5% phosphoric acid) in a 96-well microtitre plate (Maxisorb Immunoplate, Nunc). After 10 min incubation at room temperature the absorbance at a wavelength of 540 nm was measured in a microplate reader (model 450; Bio-Rad). A range of 2-fold dilutions of sodium nitrite (0.05 to100 μM) in RPMI medium was run in each assay to generate a standard curve. In most experiments, control cultures not infected or stimulated gave small nitrite values in the order of 0–1 μM. These figures were subtracted from the experimental values in the data presentation.

### Ethics

The work presented in this paper did not include research on humans. Animals, used for isolation of cells for in vitro experiments were sacrificed by cervical dislocation without receiving any prior experimental treatment. Therefore, to perform the experiments described in this article, no need for approval from ethics committees was required according to Danish law.

## Abbreviations

ARE: adenylate uridylate rich elements; AU: adenylate uridylate; CAT: chloramphenicol acetyl transferase; FCS: fetal calf serum; MAPK: mitogen-activated protein kinase; MKK: mitogen-activated protein kinase kinase; NF: nuclear factor; NOD: nucleotide-binding oligomerization domain; PAMP: pathogen-associated molecular pattern; PRR: pattern recognition receptor; RIP: receptor-interacting protein; TAK: transforming growth factor-activated kinase; TLR: Toll-like receptor; TNF: tumor necrosis factor; UTR: untranslated region.

## Competing interests

The authors declare that they have no competing interests.

## Authors' contributions

THM and SRP designed experiments. THM and RSB performed experiments. THM, RSB, and SRP interpreted data. All authors helped preparing the manuscript and approved the final version.
